# Mid-Task Break Improves Global Integration of Functional Connectivity in Lower Alpha Band

**DOI:** 10.3389/fnhum.2016.00304

**Published:** 2016-06-17

**Authors:** Junhua Li, Julian Lim, Yu Chen, Kianfoong Wong, Nitish Thakor, Anastasios Bezerianos, Yu Sun

**Affiliations:** ^1^Singapore Institute for Neurotechnology (SINAPSE), National University of SingaporeSingapore, Singapore; ^2^Neuroscience and Behavioral Disorder Program, Centre of Cognitive Neuroscience, Duke-NUS Graduate Medical SchoolSingapore, Singapore

**Keywords:** functional connectivity, lower alpha, mental fatigue, graph theoretical analysis, mid-task break, sustained attention, EEG

## Abstract

Numerous efforts have been devoted to revealing neurophysiological mechanisms of mental fatigue, aiming to find an effective way to reduce the undesirable fatigue-related outcomes. Until recently, mental fatigue is thought to be related to functional dysconnectivity among brain regions. However, the topological representation of brain functional connectivity altered by mental fatigue is only beginning to be revealed. In the current study, we applied a graph theoretical approach to analyse such topological alterations in the lower alpha band (8~10 Hz) of EEG data from 20 subjects undergoing a two-session experiment, in which one session includes four successive blocks with visual oddball tasks (session 1) whereas a mid-task break was introduced in the middle of four task blocks in the other session (session 2). Phase lag index (PLI) was then employed to measure functional connectivity strengths for all pairs of EEG channels. Behavior and connectivity maps were compared between the first and last task blocks in both sessions. Inverse efficiency scores (IES = reaction time/response accuracy) were significantly increased in the last task block, showing a clear effect of time-on-task in participants. Furthermore, a significant block-by-session interaction was revealed in the IES, suggesting the effectiveness of the mid-task break on maintaining task performance. More importantly, a significant session-independent deficit of global integration and an increase of local segregation were found in the last task block across both sessions, providing further support for the presence of a reshaped topology in functional brain connectivity networks under fatigue state. Moreover, a significant block-by-session interaction was revealed in the characteristic path length, small-worldness, and global efficiency, attributing to the significantly disrupted network topology in session 1 in comparison of the maintained network structure in session 2. Specifically, we found increased nodal betweenness centrality in several channels resided in frontal regions in session 1, resembling the observations of more segregated global architecture under fatigue state. Taken together, our findings provide insights into the substrates of brain functional dysconnectivity patterns for mental fatigue and reiterate the effectiveness of the mid-task break on maintaining brain network efficiency.

## 1. Introduction

Mental fatigue commonly occurs after a prolonged period engaging in a cognitive task (Boksem and Tops, 2008), especially in a boring and repetitive task. The ability to successfully implement a task is diminished under the condition of fatigue, exhibiting the slower response speed and the increased propensity to commit errors and lapses. In the contemporary society, mental fatigue is a prevalent problem that office workers have to face daily because of intense and stressful work. Up to now, much effort has been devoted to investigating the neurophysiological mechanism of mental fatigue (Boksem et al., 2005; Simon et al., 2011; Craig et al., 2012; Käthner et al., 2014; Sun et al., 2014). In these studies, a diversity of paradigms, ranging from trial-based sustained attention tasks, e.g., psychomotor vigilance test (Lim et al., 2012; Sun et al., 2014) to more realistic scenario, e.g., car-driving simulation (Lin et al., 2010), were employed to induce mental fatigue. Among them, sustained attention tasks have been particularly amenable to studies of mental fatigue because of their reliability and validity for inducing mental fatigue. Moreover, the neural mechanisms associated with sustained attention are fairly well understood. In our previous neuroimaging study of sustained attention (Lim et al., 2010), we found that cerebral blood flow in the fronto-parietal regions tends to decrease as time-on-task (TOT) increases, possibly reflecting a depletion of neural resources, or an inability to retrieve these resources (Langner et al., 2010).

TOT-related changes of brain electrophysiological activities have been consistently revealed, with a large number of studies showing that spectral power density was associated with the effect of prolonged task (Simon et al., 2011; Craig et al., 2012; Sauvet et al., 2014; Trejo et al., 2015). For instance, Craig et al. (2012) have reported a spectral power increase in widespread cortex when participants became fatigued in a monotonous simulated driving task. Under the fatigue state, participants paid less attention to the driving task, and showed signs of lower cognitive capacity, which significantly changed the representation of spectral power (Craig et al., 2012). Although the above-mentioned studies are informative and useful in identifying vigilance levels and characterizing spectral power attributes of mental fatigue, they have not taken into account temporal relationships between brain regions, which is crucial when the attention system is challenged. These relationships are termed by functional connectivity, referring to the activity synchronization between brain regions that are anatomically distinct but functionally collaborative. Functional connectivity in sustained attention and fatigue have only been explored in recent studies. For instance, the fronto-parietal connectivity network relevant to mental fatigue was reported in an fMRI study (Esposito et al., 2014). Liu and colleagues further reported a weakened fronto-to-parietal functional coupling in the alpha band when mental fatigue levels increased (Liu et al., 2010). This weakened fronto-parietal connection observed under fatigue might reflect functional deterioration in task implementation. Because task execution required activation in the fronto-parietal network and suppression in the default mode network, showing anti-correlation between these two networks (Gao and Lin, 2012). The competing relationship between the fronto-parietal network and the default mode network implies that the anti-correlation between them should be strong without fatigue and might be reduced under fatigue. This reduction of the anti-correlation has been observed after a prolonged attention task, showing increased functional connectivity between the posterior cingulate cortex (PCC) and middle frontal gyrus (MFG) (Gui et al., 2015). Most recently, Clayton and colleagues proposed a model of the roles of cortical oscillations in sustained attention and highlighted the role of inter-areal communication via low-frequency phase synchronization in maintaining sustained attention (Clayton et al., 2015). These studies have only investigated functional connections between regions and did not interrogate connectivity distribution (Liu et al., 2010; Gao and Lin, 2012; Esposito et al., 2014; Clayton et al., 2015). As such, they have been unable to quantitatively comment on fatigue-related alterations of connectivity topology, an analysis method that yields abundant information about brain functional segregation and integration. This assessment of functional segregation and integration is important to explore inter-regional organization as the brain is functionally separated to achieve specialized processes and is functionally assembled to integrate information through interaction and coordination. However, the investigation of connectivity topology of mental fatigue was inadequate and the knowledge of such topology change was still rudimentary (Sun et al., 2014).

Because of the undesirable outcomes caused by mental fatigue (Tucker et al., 2003), great efforts have been made to find an effective way to relieve mental fatigue. It is pervasively believed that rest is able to relieve both physical and mental fatigues and restore resource and energy for the following expenditure of task execution. There were evidences that rest break was an effective manner to maintain performance and to control accumulation of risk in a prolonged task (Tucker, 2003; Tucker et al., 2003). Although no one doubts the effectiveness of rest break to fatigue mitigation, how rest should be administered (e.g., duration, number of rest breaks) is still in debate. Furthermore, the effect of rest break to people is divergent in relieving physical fatigue and mental fatigue. For instance, the requirement of rest duration for fully relieving physical fatigue varied across people, exhibiting that the percentage of recuperative people was increased with the increasing of the rest time (Chan et al., 2012). Besides, the rest break was also effective in improving comfort and productivity of workers (Dababneh et al., 2001). For the mental fatigue relief, the restorative effect of rest is more complicated. Longer rest break was associated with greater immediate improvement in reaction time, but was followed by a steeper decrement in the subsequent performance (Lim and Kwok, 2016). The efficacy of mental break for mental fatigue mitigation was also not consistent and varied over circumstances. Ross and colleagues showed that a short break is helpful to reduce the speed of vigilance decrement, but the effect of the second short break was not significant (Ross et al., 2014). Moreover, rest was effective to relieve fatigue in a simulated driving experiment, but the impact of break was different among individuals (Phipps-nelson et al., 2011). If the break was administered through switching task (switch to a task that consumes different resource as the current task), its benefit on fatigue relief was not found (Helton and Russell, 2012). The complex effect of rest break on mental fatigue motivated us to investigate whether a rest break affects brain connectivity architecture.

The lack of adequate exploration on fatigue-related topology alteration and the unclear effect of a resting break on brain connectivity architecture motivated us to set up a visual oddball task experiment with two sessions. Four task blocks were consecutively implemented in one session (session 1) whereas a mid-task break was introduced in the middle of four task blocks in the other session (session 2). Through this experiment, we expect to address two hypotheses: (1) brain connectivity topology is altered by the effect of time-on-task and mental fatigue might lead to a less integrated connectivity architecture, expressing reduced network efficiency. (2) a mid-task break might play a role on the alteration of connectivity topology caused by fatigue, and would make a positive effect on maintaining connectivity network efficiency. As consistently reported in the power spectral density studies, the lower alpha band was closely related to mental fatigue (Oken and Salinsky, 1992; Klimesch et al., 1998; Klimesch, 1999; Boksem et al., 2005; Craig et al., 2012; Sun et al., 2014). Therefore, we directly focused on this band to explore connectivity properties in this study. Phase synchronization was utilized to construct connectivity matrices with entries represented relationships of all pairs of channels (Stam et al., 2009; Sun et al., 2012). Then, connectivity topology was analyzed by a standard graph theory framework to explore underlying connectivity architecture (Bullmore and Sporns, 2009; Rubinov and Sporns, 2010).

## 2. Materials and methods

### 2.1. Participants

Twenty right-handed students and staff members from the National University of Singapore (NUS) participated in this study. Average age was 23.1 years old (standard deviation: 3.1, range: 20 ~ 32). Twelve of them are female. All participants had normal or corrected-to-normal vision and no history of chronic physical or mental illness. They were required to ensure adequate sleep (more than 7 h) at two nights and to refrain from consuming caffeine or alcohol and not to undertake strenuous exercise within 24 h prior to the experiment. Each participant gave the written informed consent after they were clearly aware of the experiment. S$120 was paid to compensate their participation. This study was reviewed and approved by the Institutional Review Board of the NUS.

### 2.2. Experimental settings

**Figure 1:**
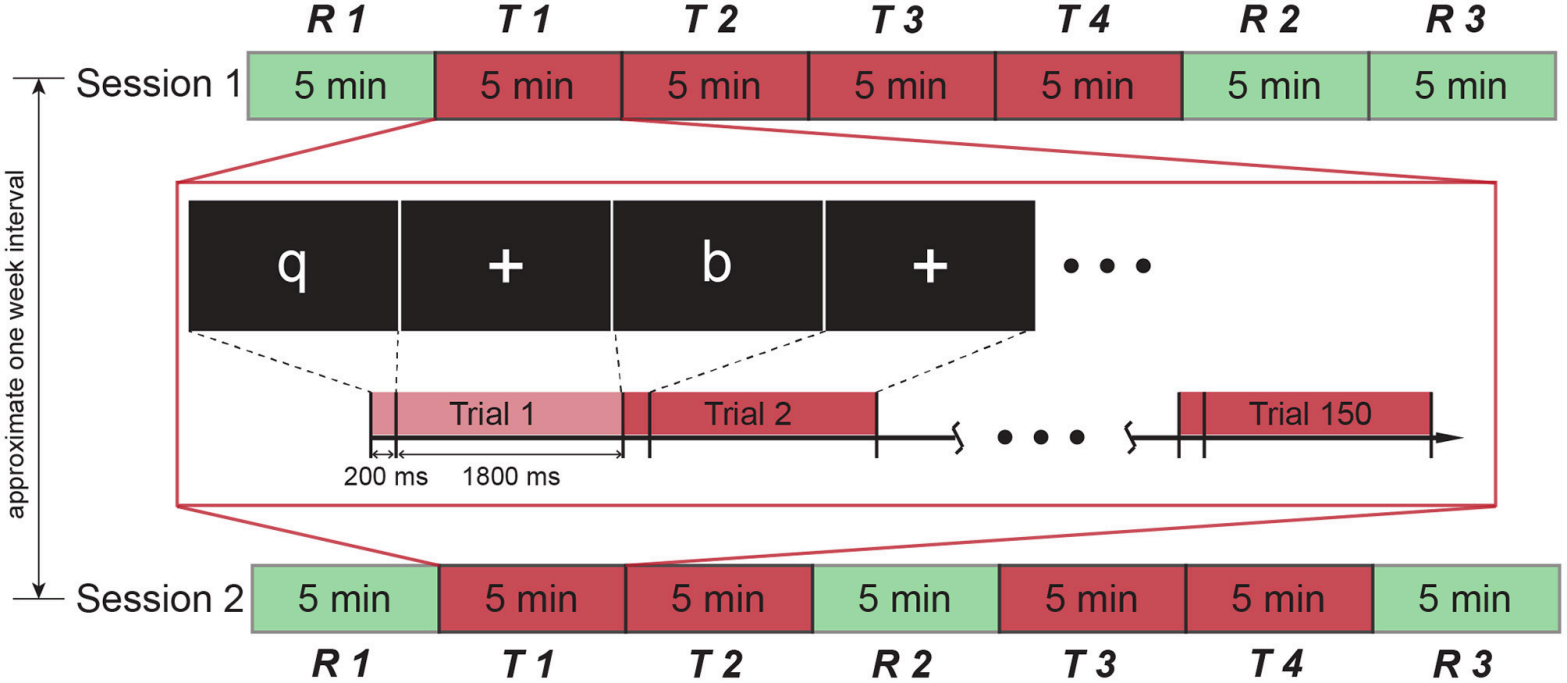
**Experiment protocol**. The experiment contained two sessions, each of which consists of 7 blocks. A resting block was presented in the middle of task blocks in session 2, while task blocks in session 1 were all successive. The interval between two sessions was approximate 1 week. The detail of the task block is illustrated in the middle rectangle. Each task block comprised 150 trials, each of which lasted 2 seconds. One of four letters (“**q**,” “**p**,” “**b**,” “**d**”) was displayed in the first 200 ms, followed by a fixation cross presentation during the remaining 1800 ms.

In this study, participants took part in two sessions with an interval of approximate 1 week between sessions. Session order was counterbalanced for participants to eliminate possible effects of session order (half participants underwent session 1 first). Each session comprised three resting blocks (*R*, 5-min long) and four task blocks (*T*, 5-min long) (see Figure 1). There was an interval of about 20 s between blocks, which allowed experimenter to reset recording. In this short time, participant kept the position and waited for the next block. Both sessions started and ended with a resting block. Four task blocks in session 1 were administered successively, while a resting block was introduced in the middle of task blocks of session 2. In the resting block, a white fixation cross was presented at the center of the screen while the participant was instructed to blink normally and refrain from moving in the scanner. A selective attention task was employed in the task blocks. In each task block, one of four alphabet letters (“**q**,” “**p**,” “**b**,” and “**d**”) was selected as target stimulus and the rest of three letters are non-target stimuli. Besides, a null stimulus was also used, which showed a blank screen without any letter. Each task block consisted of 150 trials which included target stimuli (30 trials, 20%), null stimuli (30 trials, 20%), and non-target stimuli (90 trials, 60%). For each trial, the letter was presented for 0.2 s, followed by a 1.8-s period showing fixation cross. The order of trials was randomized in each task block. The target letter differed in task blocks and the sequence of target letter in each session was also randomized for all participants. Participants were instructed to press a unique predesignated button using their right hand as quickly and accurately as possible when a target letter was presented at the center of the screen while random stimuli were being sequentially shown on the screen.

The trial sequences were generated using optseq (http://surfer.nmr.mgh.harvard.edu/optseq). Stimuli were presented using Psychtoolbox (David, 1997; Pelli, 1997) embedded in Matlab R2011a (Mathwork, USA) on a NordicNeuroLab LCD monitor 32” (NordicNeuroLab Inc., Norway) behind Siemens Magnetom Prisma scanner (Erlangen, Germany), viewed through a mirror device placed on the head coil. Behavioral responses during the MR scanning were collected using a Current Designs HHSC-2 × 4-C (Current Designs, Inc., Philadelphia) response pad through a Current Designs fORP 932 electronic interface.

### 2.3. Data acquisition

Sixty four electrodes were used for EEG recording according to the international 10–20 standard system by battery powered amplifier (Waveguard, ANT B. V., Netherlands), which was connected to the recording PC outside the scanning room through optical wires. The cable linking the Waveguard cap and the amplifier was attached to the scanner to avoid cable movements during scanning. EEG was recorded with high resolution at the sampling frequency of 4 kHz and referenced to the average of both mastoids (M1 and M2). The impedance was maintained below 15 kΩ. During concurrent EEG/fMRI recordings, participants lay quietly inside a 3T Siemens scanner and their heads were snugly fixed with foam pads to minimize head motion. fMRI data were acquired with parameters: echo planar imaging (33 slices, TR = 2 s, TE = 30 ms, slice thickness = 3.5 mm, slice-gap = 0.7 mm, flip angle = 90°, resolution = 74 × 74, in-plane resolution = 3 mm). All concurrent EEG/fMRI recordings were conducted at the Clinical Imaging Research Centre (CIRC) in Singapore. Only EEG data were included for connectivity analyses in this paper.

### 2.4. Preprocessing and functional connectivity estimation

All EEG recordings were first denoised by a customized artifact removal method (its schematic flowchart can be found in the Supplementary Figure S1). Detailed description was shown in the supplement. Briefly, EEG signals were first up-sampled to 40 kHz to facilitate volume alignment. All volumes in the same channel were then assembled into a matrix, in which rows represented volumes. Subsequently, canonical correlation analysis (CCA) (Hotelling, 1936) was utilized to seek components that maximize the correlation between volume-matrix and its one-point lag version. Those components relevant to MRI artifacts were removed and the remaining components were used to reconstruct clean EEG. Because spectral power representation during eyes-open and eyes-closed periods are prominently different in alpha band and can be consistently observed (Barry et al., 2009), this criterion was utilized to validate our method whether the critical alpha difference was retained after artifacts removal. EEG data recorded under eyes-open and eyes-closed conditions with and without scanning were used to assess effectiveness of the proposed method. Performance was also compared with the state-of-the-art artifact removal method (Optimal Basis Set, OBS) (Niazy et al., 2005) and the comparison results were shown in supplementary materials. It can be clearly seen that the proposed method was of comparable performance of the OBS. The critical phenomenon of alpha power difference was retained after artifact removal, showing suppression in alpha power when eyes were open (see Supplementary Figure S2).

After removing artifacts, EEG signals were down-sampled and band-pass filtered to the lower alpha band (8 ~ 10 Hz) using an FIR filter with the order of 660. The filtered continuous signals were partitioned into segments with 2-s length according to the onsets of stimuli, resulting in 150 segments for each task block. For each segment, phase lag index (PLI) was applied to estimate phase synchronization that are invariant against the presence of volume conduction and different montages (Stam et al., 2007). Although the PLI is relatively vulnerable to random disturbance than the weighted phase lag index (Vinck et al., 2011), this effect can be canceled out by averaging over segments. As shown in Rana et al. (2013), the PLI can perform well if the number of segments was large (e.g., 80). In our study, the number of segments is 150, which is large enough to ensure applicability of PLI. Furthermore, the PLI has been already employed by many studies to successfully detect intrinsic characteristics contained in physiological signals, such as mental deterioration (Yu et al., 2016).

Let *s*_*k*_(*t*) and *s*_*l*_(*t*) indicate time series of the *k*th and *l*th channels in a segment. Their analytical representation can be derived from the real signals *s*_*k*_(*t*) and *s*_*l*_(*t*) using the Hilbert transform as follows (Tass et al., 1998; Celka, 2007; Aydore et al., 2013)

(1) $$\begin{array}{l} \begin{array}{c} z_{k}\left(t\right)=A_{k}\left(t\right)e^{j\phi_{k}\left(t\right)}\\ z_{l}\left(t\right)=A_{l}\left(t\right)e^{j\phi_{l}\left(t\right)}, \end{array} \end{array}$$

where *A*_*k*_(*t*) and *A*_*l*_(*t*) are instantaneous amplitudes at time point *t*, and ϕ_*k*_(*t*) and ϕ_*l*_(*t*) are instantaneous phases at time point *t*. Then, instantaneous phase difference between *s*_*k*_(*t*) and *s*_*l*_(*t*) are obtained by

(2) $$\begin{array}{l} \Delta \phi_{k,l}\left(t\right)=\phi_{k}\left(t\right)-\phi_{l}\left(t\right). \end{array}$$

Finally, PLI can be calculated from a time series of phase differences Δϕ_*k, l*_(*t*_*i*_), *i* = 1…*N* by Stam et al. (2007)

(3) $$\begin{array}{l} PLI\left(k,l\right)=|\langle sign\left[\Delta \phi_{k,l}\left(t_{i}\right)\right]\rangle |, \end{array}$$

where 〈·〉 denotes the mean value, |·| denotes the absolute value, and *sign* stands for signum function. PLI value ranges from 0 to 1. A PLI value of zero means either no coupling or coupling with a phase difference centered around 0 and π and a value of one indicates perfect phase locking with consistent phase difference other than 0 and π (Stam et al., 2007). The above procedure was repeated for all pairs of channels. Subsequently, all PLIs were assembled to form a connectivity matrix, indicating by *M*. The codes for PLI implementation were from the Neurophysiological Biomarker Toolbox (http://www.nbtwiki.net/).

### 2.5. Graph theoretical analysis

A network is defined by a collection of nodes and edges. In our case, nodes represent channels and edges represent the strengths of estimated PLI. Weak and non-significant edges in a connectivity matrix may represent spurious connections (Rubinov and Sporns, 2010), so sparsity threshold, defined as the ratio of the number of actual edges to the number of all possible edges in a fully connected network, is often applied to remove those spurious connections (Rubinov and Sporns, 2010). Since there is no definitive way to determine a precise sparsity threshold (Achard and Bullmore, 2007), we explored graph metrics with a wide range of sparsity from 0.1 to 0.4 with an incremental step of 0.01, resulting in 31 sparse connectivity matrices. These sparse connectivity matrices were then binarized to obtain connectivity matrices with entry values of either 1 (connected) or 0 (unconnected). All graph theoretical metrics (shown in the next paragraph) were calculated based on these binary connectivity matrices. Finally, the integrals (an integral is equivalent to the area under the curve of that metric as a function of sparsity) of graph theoretical metrics were calculated over the range of sparsity (Achard and Bullmore, 2007). In this way, summary measures that were independent of a single threshold selection were obtained to quantitatively compare the network properties. After respectively calculating above integrals of graph metrics for each segment, these integrals for each metric were averaged across all segments belonging to the same block.

In this study, we investigated connectivity topology at both global and regional scales. Clustering coefficient (*C*) and characteristic path length (*L*) (Watts and Strogatz, 1998; Rubinov and Sporns, 2010) were utilized to explore local density and integration of connectivity network. Small-worldness (σ) (Humphries and Gurney, 2008) was employed to investigate integrated property combining high local density and short path length. Local efficiency (*El*) and global efficiency (*Eg*) (Latora and Marchiori, 2001) were used to further illustrate clear and direct physical meaning of small-wordness in terms of connective efficiency. Betweenness centrality (Freeman, 1979) was adopted to explore nodal importance at a regional scale. Definitions and brief descriptions of these metrics were listed in the Table 1. Metric calculation was implemented by using the codes from the brain connectivity toolbox (Rubinov and Sporns, 2010).

**Table 1:** **Definitions and brief descriptions of the network metrics applied in this study**.

**Network metrics**	**Definitions**	**Brief descriptions**
**GLOBAL METRICS**		
Clustering coefficient (*C*)	$C=\frac{1}{n}\underset{i\;\in \;N}{\sum }\frac{\underset{j,h\in N}{\sum }m_{ij}m_{ih}m_{jh}}{k_{i}\left(k_{i}\;-\;1\right)}$	*C* is the average of clustering coefficients of all nodes, reflecting the prevalence of clustered connectivity around individual nodes. The clustering coefficient of node *i* is the fraction of triangles around this node and is equivalent to the fraction of node's neighbors that are neighbors of each other. *k*_*i*_ is the number of edges connected to the node *i*, *m*_*ij*_ is a binary value indicating connection status (*m*_*ij*_ = 1, connected; *m*_*ij*_ = 0, disconnected), and *N* stands for the set of *n* nodes.
Characteristic path length (*L*)	*L* = $\frac{1}{n}\underset{i\;\in \;N}{\sum }\frac{\sum_{j\in N,\;j\;\neq \;i}d_{ij}}{n\;-\;1}$	*L* is defined as the average shortest path length between all pairs of nodes in a network, measuring functional integration (how well integrated a network is). *d*_*ij*_ is the shortest path length between nodes *i* and *j*.
Small-worldness (σ)	$\sigma =\frac{\gamma }{\lambda }=\frac{C∕C_{rand}}{L∕L_{rand}}$	σ is defined as the ratio of normalized clustering coefficient γ to normalized characteristic path length λ. γ is a ratio of the clustering coefficient *C* of the tested network to the mean of clustering coefficients *C*_*rand*_ of 100 surrogate random networks. These random networks were generated from the original network by randomly reshuffling edges while preserving basic characteristics of size, density and degree distribution of the original network. λ is a ratio of *L* to the mean of characteristic path lengths *L*_*rand*_ of 100 surrogate random networks.
Global efficiency (*Eg*)	$Eg=\frac{1}{n}\underset{i\;\in \;N}{\sum }\frac{\sum_{j\in N,\;j\;\neq \;i}d_{ij}^{-1}}{n\;-\;1}$	*Eg* is the average inverse shortest path length in the network, which is closely related to *L*.
Local efficiency (*El*)	$El=\frac{1}{n}\underset{i\;\in \;N}{\sum }\frac{\sum_{j,\;h\in N_{i},\;j\;\neq \;h}{\left[d_{jh}\left(N_{i}\right)\right]}^{-1}}{k_{i}\left(k_{i}\;-\;1\right)}$	*El* is the global efficiency only computed on node neighborhoods. *d*_*jh*_(*N*_*i*_) is the length of the shortest path between *j* and *h* that are in the neighborhoods (the set of nodes that directly connect to the node *i*) of node *i*.
**LOCAL METRIC**		
Betweenness centrality (*BC*)	BCi=1(n - 1)(n-2)∑h,j∈Nh ≠ j ≠ iphj(i)phj	*BC* measures how important a node exerting in a network, which defines as the fraction of all shortest paths in the network that contain this node. *p*_*hj*_ is the number of shortest paths between nodes *h* and *j*, and *p*_*hj*_(*i*) is the number of shortest paths between nodes *h* and *j* that pass through the node *i*.

### 2.6. Statistical analysis

Linear mixed model (LMM) (Krueger and Tian, 2004) with block and session as fixed effects and subject label as random effect was used to compare means of behavioral measures and connectivity metrics to determine whether there were statistically significant effects of blocks [two levels: the first task block (T1) and the fourth task block (T4)], sessions (two levels: session 1 and session 2), and block × session interaction. The *post-hoc* two-tailed paired *t*-test was subsequently conducted on paired samples to check whether there was significant difference between their means in both directions if a significant main effect was found in the LMM analysis. A value of *p* < 0.05 was considered significant. Because of the exploratory nature of the current study, corrections for multiple testing were not applied.

## 3. Results

### 3.1. Behavioral data analysis

**Figure 2:**
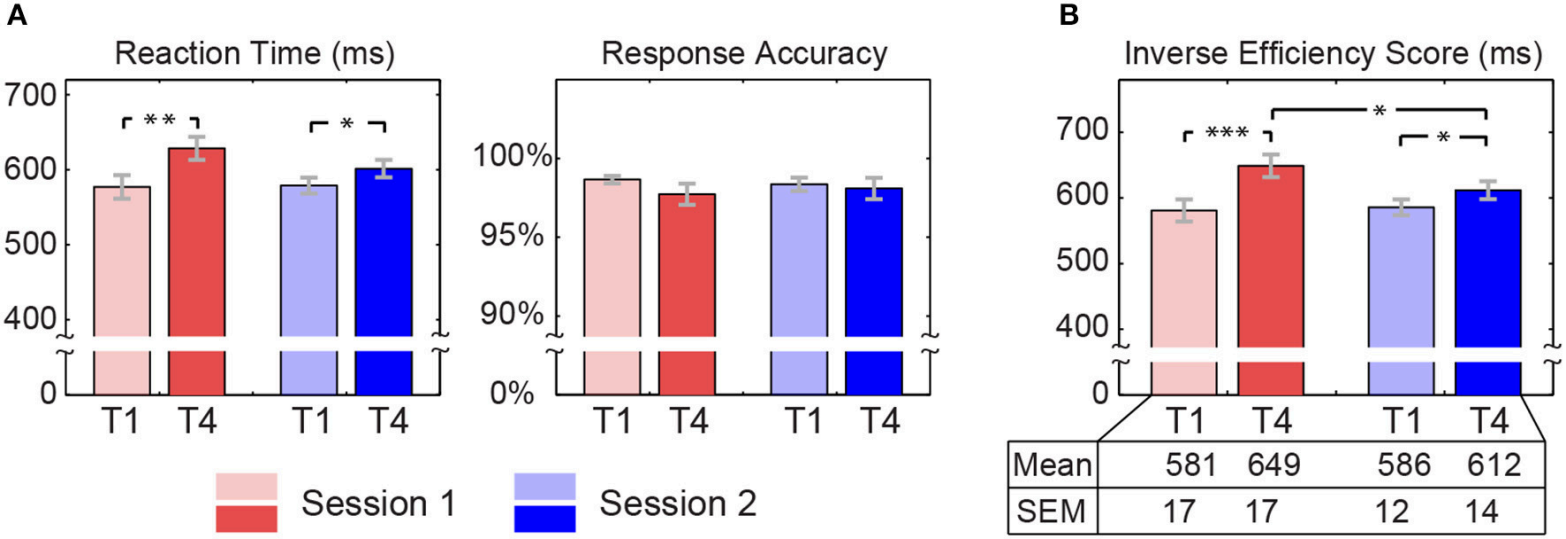
**Behavioral data**. Inverse efficiency score is derived from that average reaction time within a task block is divided by response accuracy of that task block, which was calculated for each subject. Red bars represent average behavioral measures across subjects for session 1, while blue bars are for session 2 (light colors indicate the first task block and dark colors indicate the fourth task block). Gray error bars represent standard errors. Asterisks at the top indicate those pairs with statistically significant difference by the *post-hoc* t-test (^*^p < 0.05, ^**^p < 0.005, ^***^p < 0.0005). The table at the bottom lists means and standard errors (SEM) of inverse efficiency score.

Reaction time in both sessions were increased from T1 (Session 1: 577 ± 16 ms; Session 2: 579 ± 11 ms) to T4 (Session 1: 629 ± 15 ms; Session 2: 602 ± 12 ms). The increasing extent was larger for session 1 than that for session 2. Generally speaking, all participants performed well with high rate of correct responses in each block of both sessions. Although response accuracy was high in each block, we still observed a decline trend from T1 (Session 1: 98.67% ± 0.24%; Session 2: 98.37% ± 0.43%) to T4 (Session 1: 97.73% ± 0.68%; Session 2: 98.10% ± 0.68%). We further estimated the inverse efficiency score to scale the extent of fatigue, which dismiss possible criterion bias or speed accuracy trade-off (Jacques and Rossion, 2007). This inverse efficiency score was derived from that the mean of reaction time averaged within a task block divided by the response accuracy of that block. The LMM analyses revealed that there was a significant block effect on reaction time [T1 < T4, *F*_(1, 57)_ = 19.223, *p* < 0.001] and the inverse efficiency score [T1 < T4, *F*_(1, 57)_ = 27.319, *p* < 0.001]. More interestingly, a significant block × session interaction was revealed in the inverse efficiency score [*F*_(1, 57)_ = 5.134, *p* = 0.027]. Further *post-hoc* test showed that the significant interaction was attributed to a highly significant increase of the inverse efficiency score in session 1 [*t*_(19)_ = −4.590, *p* < 0.001] in comparison to a significant increase in session 2 [*t*_(19)_ = −2.549, *p* = 0.020] (see Figure 2). No significant block effect, session effect, and interaction (*p* > 0.05) were found in response accuracy.

### 3.2. Global network properties

**Figure 3:**
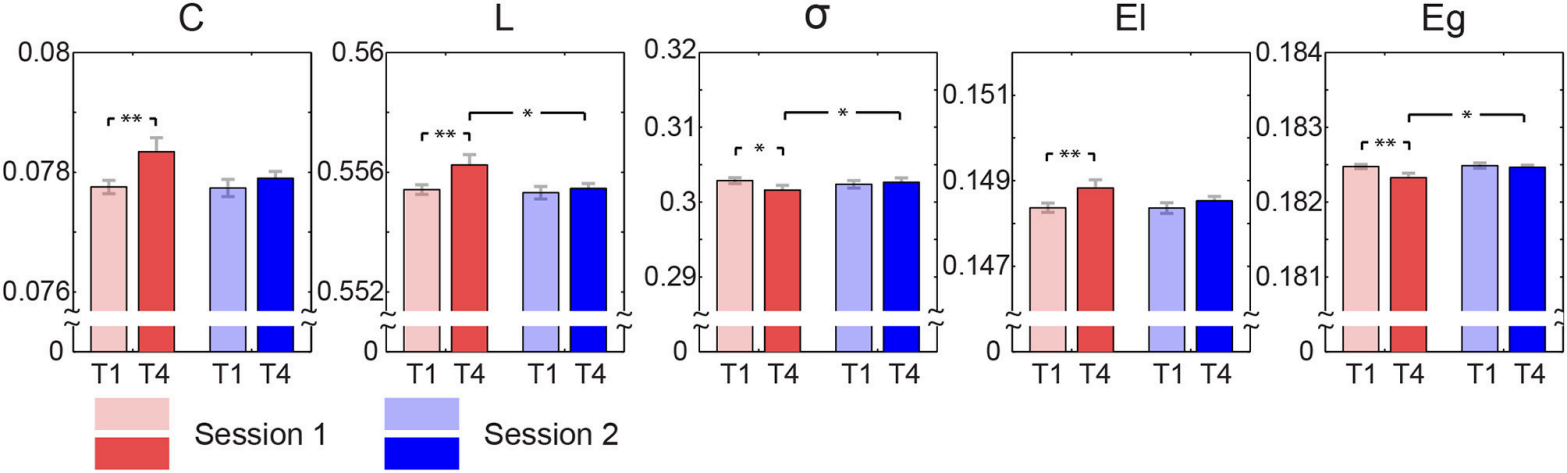
**Means and standard errors of graph metrics of functional connectivity**. Bars represent means averaged across subjects and error bars indicate corresponding standard errors. Asterisks are used to mark those pairs with statistically significant difference (^*^p < 0.05, ^**^p < 0.005).

Quantitative statistical analyses revealed significant topological changes in the global network metrics between both blocks and sessions, which were conducted by comparing blocks (T1 and T4) and sessions (Session 1 and Session 2). The results derived from the LMM statistical analyses were listed in Table 2. A significant block effect was found in clustering coefficient [T1 < T4, *F*_(1, 57)_ = 9.806, *p* = 0.003], characteristic path length [T1 < T4, *F*_(1, 57)_ = 8.600, *p* = 0.005], local efficiency [T1 < T4, *F*_(1, 57)_ = 8.283, *p* = 0.006], and global efficiency [T1 > T4, *F*_(1, 57)_ = 8.916, *p* = 0.004]. Significant session effect was observed in characteristic path length [*Session*1 > *Session*2, *F*_(1, 57)_ = 7.394, *p* = 0.009] and global efficiency [*Session*1 < *Session*2, *F*_(1, 57)_ = 6.928, *p* = 0.011]. More interestingly, significant block × session interaction was discovered on characteristic path length [*F*_(1, 57)_ = 4.324, *p* = 0.042], small-worldness [*F*_(1, 57)_ = 5.219, *p* = 0.026] and global efficiency [*F*_(1, 57)_ = 4.695, *p* = 0.034]. The *post-hoc* two-tailed paired *t*-test revealed that these interactions were resulted from significant change from T1 to T4 for session 1, but not for session 2 (see Figure 3).

**Table 2:** **Statistical analyses results of graph theoretical metrics**.

**Metrics**	**Block effect *F*_(1, 57)_[*p*-value]**	**Session effect *F*_(1, 57)_[*p*-value]**	**Interaction *F*_(1, 57)_[*p*-value]**
*C*	**9.806 [0.003]**^▴^	3.645 [0.061]	3.109 [0.083]
*L*	**8.600 [0.005]**^▴^	**7.394 [0.009]**^↓^	**4.324 [0.042]**
σ	2.081 [0.155]	0.743 [0.392]	**5.219 [0.026]**
*El*	**8.283 [0.006]**^▴^	1.979 [0.165]	1.784 [0.187]
*Eg*	**8.916 [0.004]**^▾^	**6.928 [0.011]**^↑^	**4.695 [0.034]**

*Boldface indicates that there is a statistical significance in a comparison*.

▴ T1 < T4;

▾ T1>T4;

↑ Session1 < Session2;

↓ *Session1>Session2*.

### 3.3. Nodal metric results

Using LMM statistical analyses, we further localized important nodes showing statistical mental fatigue related alterations (Table 3). Specifically, significant block effect (T1 < T4, *p* < 0.05) was revealed in four channels mainly resided in the frontal area (i.e., Fp2, FC1, FC6, and F5). Statistically significant session main effect was found in six EEG channels (including Fp1, F4, O2, AF3, CP4, and P6) distributed in the frontal region and right parietal areas. Furthermore, a significant block × session interaction was revealed in the PO8 channel, attributing to a significant reduction of betweenness centrality in session 1 and no significant change in session 2. Detailed *post-hoc* results could be found in the Supplementary Figure S3.

**Table 3:** **Comparison results of the betweenness centrality**.

**Channel**	**Block effect *F*_(1, 57)_[*p*-value]**	**Session effect *F*_(1, 57)_[*p*-value]**	**Interaction *F*_(1, 57)_[*p*-value]**
Fp1	-	6.628 [0.013]^↑^	-
Fp2	4.328 [0.042]^▴^	-	-
F4	-	7.949 [0.007]^↓^	-
FC1	4.153 [0.046]^▴^	-	-
FC6	4.017 [0.0498]^▴^	-	-
O2	-	5.897 [0.018]^↓^	-
AF3	-	5.738 [0.020]^↑^	-
F5	5.553 [0.022]^▴^	-	-
CP4	-	4.490 [0.039]^↓^	-
P6	-	4.094 [0.048]^↓^	-
PO8	-	-	6.388 [0.014]

*The statistical results were obtained by the LMM analyses with main effects of Session (session 1 and session 2) and Block (T1 and T4), and random effect of subject label*.

▴ T1 < T4;

↑ Session1 < Session2;

↓ *Session1>Session2*.

**Figure 4:**
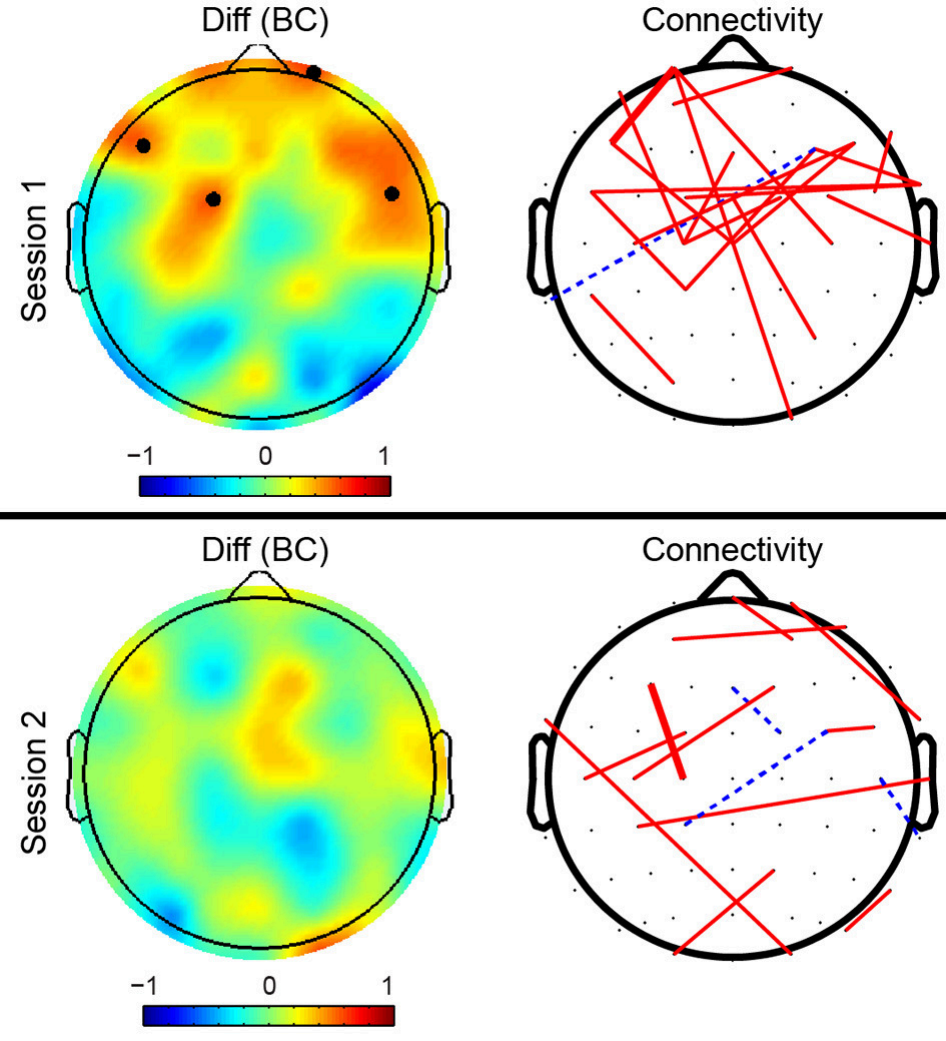
**Topographies of betweenness centrality and connectivity differences between the fourth task block (T4) and the first task block (T1)**. The left column of topographies shows the betweenness centrality differences on each node (*T*4−*T*1). Black dots indicate those nodes that have a significant block effect (LMM at the level of 0.05) and also have a significant difference between T4 and T1 (*post-hoc* two-tailed paired *t*-test at the level of 0.05). The right column shows average differences of synchrony connectivity between T4 and T1 (*T*4−*T*1) over subjects. The connectivity edges shown in the right topographies are the top 30% differences that present in more than half number of subjects (> 10). The width of lines encodes connectivity strength (the wider the line, the stronger the connectivity between them). Solid red lines indicate that connectivity in T4 was stronger than that in T1, while dashed blue lines indicate weaker connectivity in T4.

To better reveal the mental fatigue related topography alterations of brain network, we further investigated the connectivity difference between T4 and T1 in both sessions (see Figure 4). The left column in Figure 4 depicts the betweenness centrality (BC) difference between T4 and T1 and black dots highlight those nodes that showed both significant block effect (LMM analysis) and significant difference between T4 and T1 (*post-hoc* analysis). For better illustrative purposes, dominantly different connections that belonged to the top 30% of connections in difference between T4 and T1 and shared with at least half of all subjects were shown in the right column of Figure 4. The width of lines indicates difference strength. Solid red lines indicate that the strength of connections in T4 was greater than that in T1, while dotted blue lines show the opposite case. As shown in the connectivity topographies, connectivity edges with dominant difference for session 1 were largely localized in the frontal cortex, while the distribution of dominantly different edges for session 2 was more widespread and those edges were distributed over the entire cortex. The distribution patterns of dominant connections were matched with that of betweenness centrality, exhibiting consistent localization in frontal cortex in session 1 and no obvious localization in session 2.

## 4. Discussions

In this study, we utilized the graph theoretical analyses to investigate brain connectivity topology alterations caused by fatigue and the effect of a mid-task break on the fatigue-related changes in connectivity topology. A block effect was observed on inverse efficiency score, indicating the effect of TOT. Moreover, there was a significant interaction effect on the inverse efficiency score, reflecting that a mid-task break played a role on mitigation of the effect of TOT. According to the results of global metrics, fatigue led to a more segregated and less integrated connectivity network and this alteration in the network can be reduced by the mid-task break. Betweenness centrality results showed that important nodes were localized in frontal cortex under fatigue state whereas this localization was not appeared when the mid-task break interrupted the task blocks.

### 4.1. Effect of mid-task break on behavior

We found a significant main block effect on the inverse efficiency score, showing that there was significant effect of the TOT. Significant interaction was also found, which reflected that the change of inverse efficiency score from T1 to T4 depended on the setting of session. This revealed that a mid-task break had significant effect on performance. The *post-hoc* analyses showed that the inverse efficiency score was elevated from T1 to T4, implying a decline in the performance. We observed significant increasing in reaction time and non-significant change in response accuracy from T1 to T4. The significant increasing in reaction time without significant increasing in response accuracy suggests a genuine reduction in the capacity for timely responding as opposed to a speed-accuracy tradeoff. With the mid-task break, the inverse efficiency score was still significantly elevated from T1 to T4, but the increasing extent between means of T1 and T4 was shrunk in comparison with that in session 1 (no mid-task break). This might be attributed to short duration of break. The effect caused by the task was not completely eliminated within this short break. An fMRI study concluded that the effect caused by a task can be still observed after 12 min of the task completion for some participants (Breckel et al., 2013). Longer break could further counteract task effect and eliminate the difference between T1 and T4. There was evidence showing that rest duration was closely related to physical fatigue recovery and the recovery percentage of people was increased with the increase of rest time (Chan et al., 2012). Another possible cause resulting in significantly different reaction time may be due to variance between subjects. Individual differences in response to the break has been previously reported in an experiment of auditory oddball task (Lim et al., 2013). Inter-subject difference could be derived from differences in the rate of resource recovery, possibly due to the engagement of different cognitive processes during the break (e.g., rumination). When the resources engaged during the rest periods overlaps with resource requirement in the task block, rest may be less effective in counteracting the fatiguing effects of task performance (Helton and Russell, 2015).

### 4.2. Topological alterations of connectivity network

Topological properties changed with time engaged in the task. The clustering coefficient and characteristic path length were significantly increased over time in session 1, reflecting that brain regions were more segregated and communicated with each other less efficiently. This could be derived from the fact that the interactions in local neural population were enhanced to resist efficiency decline, but the communications between populations were partially suppressed, requiring longer transits for information delivery. Mid-task breaks were helpful in mitigating this effect, with lower increases in both clustering coefficient and characteristic path length observed in session 2. In the previous study, a significant increase in path length was reported (Sun et al., 2014), which is in line with the finding in this study. However, they did not find the significant change in clustering coefficient. This might be due to different methodologies employed for constructing connectivity network. In this study, connectivity network was obtained based on phase synchronization that measured instantaneous phase difference, rather than based on partial directed coherence that measured a ratio of the outflow of node *A* toward node *B* to all outflows from node *A*. Local efficiency and global efficiency are closely related to clustering coefficient and characteristic path length, respectively (Latora and Marchiori, 2001). Global efficiency is the average inverse shortest path length, preferably characterizing integration of connectivity network. Fatigue gave rise to an increase of local efficiency and a decrease of global efficiency, implying that brain resources might be reorganized and the concerted activities within regions were more active, but interactions between regions were inhibited. The disruption of short paths in the functional connectivity network resulted in lower global efficiency. In order to compensate for the lack of efficiency, individual cortical regions would exert a stronger influence, so that local efficiency was largely improved. However, due to reduced global efficiency, enhanced local efficiency was not sufficient to maintain optimal functioning, which presumably accounts for the decline in task performance.

### 4.3. Regional alterations under mental fatigue

Nodes with significant block effect on betweenness centrality were located in frontal region, suggesting that these nodes exerted more importantly in brain connectivity network with the state of fatigue. The connectivity edges that were dominantly different between T4 and T1 were relatively localized in the frontal region in session 1, while the edges with dominant difference were widespread when the mid-task break was introduced at the middle of the task blocks (see the right column in Figure 4). A more concentrated distribution under fatigue would lead to disruption in some inter-regional pathways that functioned for task execution. This finding is in agreement with the result that connection disruption was associated with fatigue in multiple sclerosis disease (Sepulcre et al., 2009). Inter-regional connections were also necessary in the attention network for coordinating attention-related subregions, especially the fronto-parietal synchronization (Clayton et al., 2015). It has been found that reductions in fronto-parietal functional connectivity were associated with cognitive fatigue (Liu et al., 2010; Sun et al., 2014). The widespread distribution of connectivity might be helpful for task implementation, which may play an important role in performing a “refresh” of processing modules so as to enhance sensitivity of processing of a subsequent stimulus (Sadaghiani et al., 2010). Interestingly, the distribution of nodes with large change in betweenness centrality was not symmetric between brain hemispheres. Such asymmetry has also been reported in other fatigue research (Sun et al., 2014). In addition, the fatigue related area observed in our study overlapped the ventrolateral prefrotal cortex that showed associations with fatigue (Suda et al., 2009).

### 4.4. Considerations and future work

A few considerations were addressed as follows. Firstly, we did not find significant correlation between behavioral measures and network metrics. We speculated that the lack of significant correlation was probably due to subject variance and the relatively small sample size. Subjects might experience different amounts of mental resource depletion in task execution and might differ in the recovery speed from mental fatigue after the task due to individual differences in cognitive capacities. This individual differences was supported by an fMRI study, showing mental resilience in brain connectivity organization is different for subjects (Breckel et al., 2013). It can lead to different changes in network metrics for different subjects. Besides, it was reported that inter-target interval affected brain activity. The increasing in inter-target interval led to increasing in brain activity over widespread cortex (Breckel et al., 2011). These causes might give rise to an outcome that the changes in behavioral measures did not well linearly match with the changes in network metrics. This dramatically reduced correlation between behavioral measures and network metrics. The relationship between behavioral measures and network metrics should be further clarified with more subjects in the future work. Secondly, statistical analyses revealed that the difference of betweenness centrality was mainly localized in the frontal cortex, part of which was close to eyes. EEG signals measured from the area near eyes are susceptible to electrooculogram (EOG) artifacts generated from eye movements. Great care was taken to minimize the influence of EOG on network analysis. Specifically, according to visual inspection of the cleaned EEG data randomly selected from half of the subjects, we did not find the sign of EOG artifacts. Furthermore, we found that the distinct difference of betweenness centrality appeared in the entire frontal region, and its distribution was asymmetric. This asymmetric pattern was not matched with the typical appearance of symmetry resulting from EOG. In the session 2, we did not observe frontal localization, which further indicated that the frontal localization in session 1 was not derived from EOG. Thirdly, as our study is an exploratory investigation of mental fatigue related alterations in brain networks, an uncorrected *p*-value of 0.05 was employed for establishing the significance and presenting the results. It is possible that some of the regional results may have occurred by chance and some cautions are needed when interpreting these results. In the current study, we focused primarily on the interpretation of the general pattern of the findings. Hence, we listed all exact statistic values along with the reported findings and left them for reader's interpretation. Fourthly, volume conduction is an issue to sensor-space EEG analysis. In order to largely exclude impact of volume conduction, the PLI that is less sensitive to volume conduction was utilized in this study to estimate connectivity strengths between brain regions. More recently, a variant of the PLI was developed for estimating phase synchronization (namely weighted phase lag index) (Vinck et al., 2011), but it is more conservative than PLI, and might underestimate true connectivity strengths by assigning smaller weights for those genuine connections with low phase differences. As pointed out in (Hillebrand et al., 2012), weighted phase lag index introduces an arbitrary bias favoring large phase differences and mixing of the estimation of consistency of phase differences with the estimation of the magnitude of the phase difference. Moreover, graph metrics based on the weighted phase lag index seem to be slightly less reliable than that of the PLI, and have a higher inter-subject variability (Hardmeier et al., 2014). However, the PLI is relatively vulnerable to random disturbance compared to the weighted phase lag index, so that the latter method is more suitable for synchronization estimation when the number of sample segments is not large enough to diminish the effect from random disturbance. Finally, only EEG data were used in this paper to explore the mental fatigue related brain network alteration. Relatively low spatial resolution of the EEG did not allow us to precisely localize fatigue-related regions. Most recently, using spontaneous activity of resting-state fMRI data, Gui et al. (2015) revealed several brain regions in the default mode network that are vulnerable to mental fatigue. The complementary advantages of EEG and fMRI modalities can be utilized to make joint inferences (Ritter and Villringer, 2006). More importantly, incorporating the explorations of both fMRI and EEG data would provide added information and more comprehensive insights into the neurophysiological mechanism of mental fatigue. The exploratory results in the current work can render heuristic clues to guide the further investigations in source space (Vecchio et al., 2015) and cross-modality analyses in a future work.

## 5. Conclusion

We designed a visual oddball task experiment to investigate the connectivity topology associated with mental fatigue and the effect of a mid-task break on topology alteration of mental fatigue. From our preliminary exploration, brain connectivity topology was changed as increasing of time-on-task, exhibiting that the functional connectivity network had more segregated and less integrated representation under mental fatigue. This fatigue-related alteration in connectivity topology can be mitigated by a mid-task break. Betweenness centrality results showed that important nodes were localized in frontal cortex under fatigue state. The frontal cortex localization was diminished when a mid-task break was introduced in the middle of task blocks. In summary, functional connectivity topology was altered due to mental fatigue and the mid-task break mitigated the extent of topology alteration. Our findings might contribute to the understanding of the effect of a mid-task break on brain topological organization and add to our knowledge of the cognitive neuroscience of work and rest.

## Author contributions

Junhua Li, Julian Lim, and YS conceived and initiated the analysis study in this paper. KW conducted the experiment and collected the data. Junhua Li, YC, and YS analyzed the data. Junhua Li, Julian Lim, YS interpreted the results and drafted the manuscript. All authors reviewed the manuscript and approved the final version for the publication.

### Conflict of interest statement

The authors declare that the research was conducted in the absence of any commercial or financial relationships that could be construed as a potential conflict of interest.
