# Supplementary material for: Mid-Task Break Improves Global Integration of Functional Connectivity in Lower Alpha Band
Source: Front Hum Neurosci. 2016 Jun 17;10:304. doi: 10.3389/fnhum.2016.00304 (PMC4911415; doi:10.3389/fnhum.2016.00304)
Supplement: Supplementary file 1 [file Image1.PDF]

frontiers

Supplementary Material

Mid-task Break Improves Global Integration of Functional Connectivity in Lower Alpha Band

Junhua Li, Julian Lim, Yu Chen, Kianfoong Wong, Nitish Thakor, Anastasios Bezerianos, Yu SUN*

*** Correspondence:** Yu SUN: lsisu@nus.edu.sg

1 Procedure of Artifact Removal

MRI artifacts embedded in EEG can vary across channels depending on the locations of the electrodes and the wire connections, so artifact removal should be implemented separately for each channel. Because the internal clock in the EEG acquisition system is independent to the clock in the MRI scanner, EEG sample timing is not well synchronized with MRI imaging sequence. This causes a difference between the real volume starting point and the labelled marker in EEG signal. Besides, the repetition time of the MRI sequence is usually not an exact multiple of the EEG sampling interval, which results in a jitter between volumes. The misalignment issue requires to first align all volumes before further processing. We up-sampled to 40 kHz in order to narrow the sampling interval, so that the alignment can be done more precisely. Because all channels share the same timing, the timing aligned on any channel can be directly applied to the rest of channels. The centre channel Cz was used in our processing for timing alignment. Radio frequency pulse, a large peak, was a typical symbol of the beginning of a volume. We referenced the first original volume marker in EEG signal to seek the nearest radio frequency pulse and temporarily determined the position ahead of radio frequency pulse as the starting point of the first volume.

In our experiment, a volume should theoretically have 80000 data points due to the setting of two-second repetition time, but there was slight bias error in practice. Therefore, a series of volume lengths varying from 79970 data points to 80010 data points were used to seek optimal volume length. The final starting point and volume length of the first volume were determined by maximizing the correlation coefficient between the first and the second volumes. This determined volume length was used for all following volumes, and rough starting points of all following volumes were estimated according to the starting point of the first volumes. Their precise starting points were sought within a specified span (i.e., 50 data points) according to correlation between successive volumes. After every ten volumes, the current volume was put into a reference pool. Correlation was conducted not only between successive volumes but also with volumes in the pool and average correlation coefficient was utilized to determine the starting point, which ensured that strong correlation was not only for local volumes, but also for volumes distant in time. Exemplary volumes (portions around radio frequency pulse) before and after alignment were illustrated in Figure S1 (a).

All volumes of one channel were assembled to construct a matrix X with rows representing volumes and columns representing data points (see Figure S1 (b)). We then constructed a one data point lag version of X, denoted by Y. The artifacts contained in these two versions was supposed to be same pattern in both shape and amplitude. We sought a projected space that transformations of X and Y can be maximally correlated. After the projection, their sources were obtained. Similarly, the rest projected spaces can be sequentially obtained based on that current source pair was maximally correlated each other and was not correlate with all previous source pairs. After all source pairs were obtained, we calculated standard deviations of sources and normalized them by the sum of standard deviations of all sources. The sources were removed if their normalized standard deviation exceeded a specified threshold (empirically set to 0.05). Gradient artifact-free EEG can be subsequently reconstructed by using only the remaining sources. The above procedure was repeated for all channels.

The EEG data of eyes-open and eyes-closed states were used for effectiveness validation of methods, because spectral power representation during eyes-open and eyes-closed periods are prominently different in alpha band and can be consistently observed (Barry et al., 2009). EEG data were recorded when participant's eyes were either open or closed without scanning. The same length EEG data were recorded for the eyes-open and eyes-closed while scanning. Performance was also compared with the state-of-the-art method (Optimal Basis Set, OBS) (Niazy et al., 2005) and the comparison results were shown in Figure S2. It can be seen that the proposed method was of comparable performance to the OBS. The phenomenon of alpha power difference was retained, showing suppression in alpha power when eyes were open.

References

Barry, R.J., Clarke, A.R., Johnstone, S.J., and Brown, C.R. (2009). EEG differences in children between eyes-closed and eyes-open resting conditions. *Clin. Neurophysiol.* 120(10)**,** 1806-1811.

Niazy, R., Beckmann, C., Iannetti, G., Brady, J., and Smith, S. (2005). Removal of fMRI environment artifacts from EEG data using optimal basis sets. *Neuroimage* 28(3)**,** 720-737.

2 Supplementary Figures

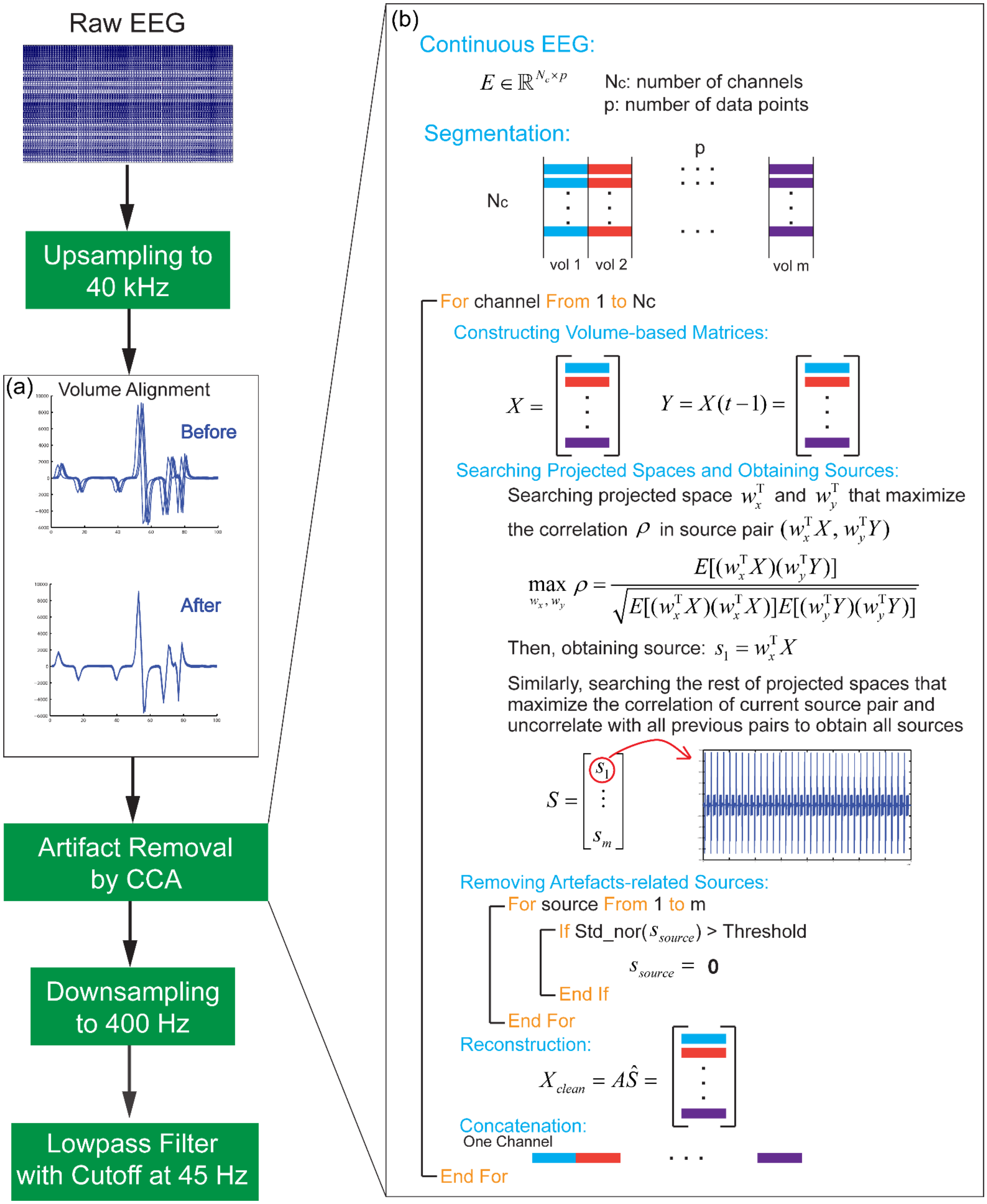

Figure S1 The schematic flowchart of the proposed CCA-based method. Firstly, raw EEG data were up-sampled to 40 kHz for facilitating volume alignment. Then, sources were obtained by CCA and artifact-related sources were removed to gain gradient artifact-free EEG. Finally, downsampling and lowpass filter were applied to reduce storage size and aliasing. (a) Illustration on a few exemplary volumes before and after the alignment. (b) The procedure of artifact removal by CCA.

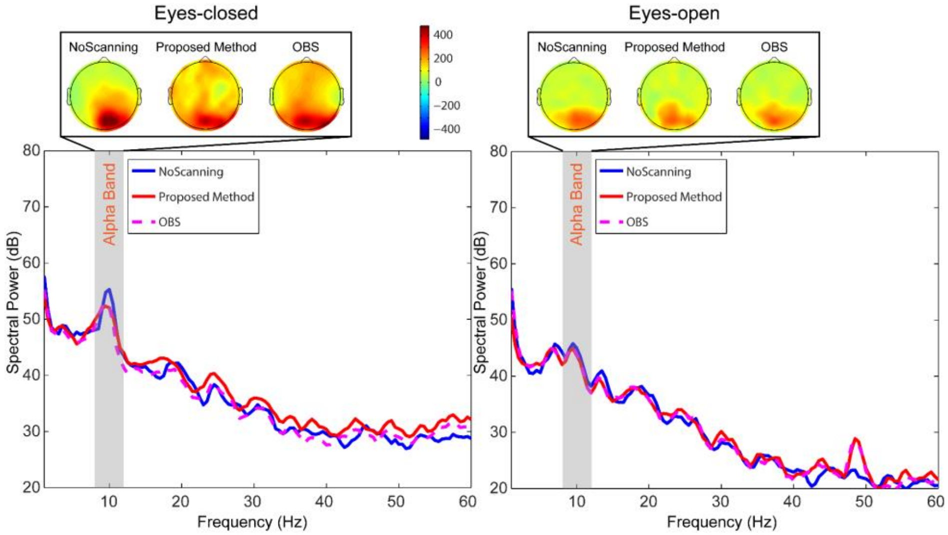

Figure S2 Comparison results. The bottom plots show power spectral densities of EEG recorded without scanning and corrected EEG after artifact removal by methods on an illustrative channel POz. The upper topographies show the spatial distribution of alpha band power with all channels.

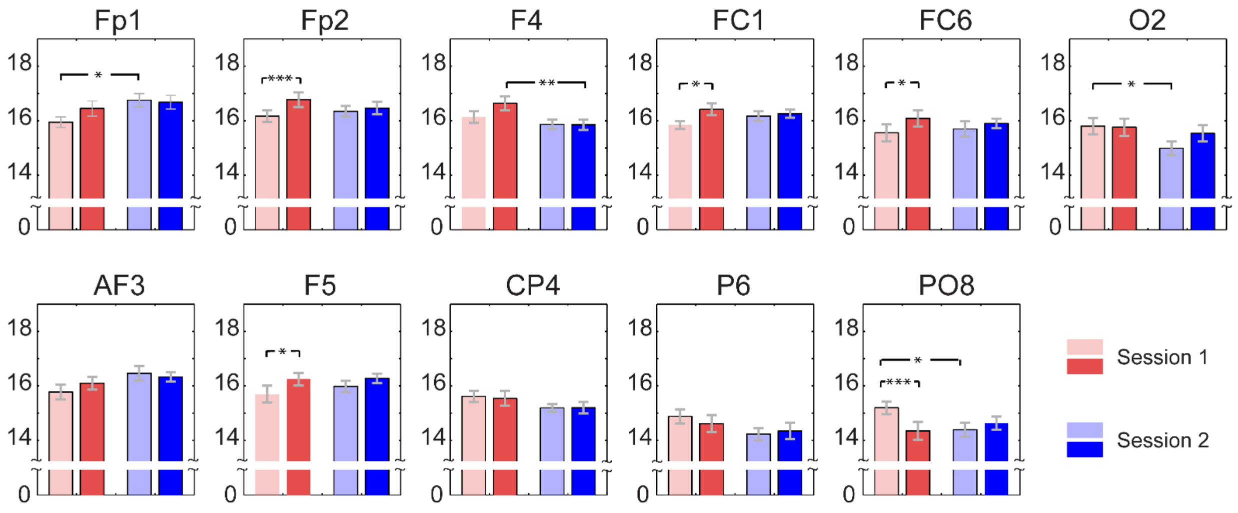

Figure S3 Means and standard errors of nodal betweenness centralities. Bars represent means averaged across subjects and error bars stand for corresponding stand errors. Asterisks indicate those pairs with statistically significant difference (* $p < 0.05$, ** $p < 0.01$, *** $p < 0.005$).
